# Case report: Documentation of cutaneous only pemphigus vulgaris without history of mucosal lesions in North America

**DOI:** 10.3389/fimmu.2022.969279

**Published:** 2022-09-08

**Authors:** John Baker, Kristina Seiffert-Sinha, Animesh A. Sinha

**Affiliations:** Department of Dermatology, Jacobs School of Medicine and Biomedical Sciences, University at Buffalo, Buffalo, NY, United States

**Keywords:** autoantibody, cutaneous, mucosal, North America, pemphigus vulgaris

## Abstract

**Background:**

Pemphigus is a group of autoimmune blistering diseases including Pemphigus vulgaris (PV) and Pemphigus foliaceus (PF). These conditions exhibit lesions with mucosal or mucocutaneous (PV) or cutaneous (PF) morphology, as framed by the Desmoglein Compensation Hypothesis (DCH). However, some PV patients present with solely cutaneous disease (cPV), and growing evidence suggests the existence of a cPV subtype *without any history* of mucosal erosions/blisters (cPVwohm), neither of which are predicted by the DCH.

**Methods:**

Participants were recruited from several outpatient clinical settings and patient support group meetings throughout the US. On intake, subjects provided blood samples and completed questionnaires regarding their disease status.

**Results:**

We report three cases of clinically and histologically confirmed cPV without history of mucosal lesions (cPVwohm). Of these patients, two do not carry the most common PV associated HLA alleles, DRB1*0402 or DQB1*0503. The same two patients also tested negative for the primary PV associated autoantibodies, anti-desmoglein 3 and anti-desmoglein 1, while in active disease status.

**Conclusion:**

We confirm the first documented individual cases of cPVwohm in North America, supporting the existence of PV patients that develop cutaneous disease without a history of mucosal lesions, challenging the fidelity of the DCH. Two of the 3 patients reported did not type for the common PV-associated HLA genes or display anti-desmoglein autoantibodies while in active disease, suggesting cPV patients may develop Pemphigus *via* genetic and immune mechanisms that differ from typical mucosal or mucocutaneous PV.

## Introduction

Pemphigus is a group of rare autoimmune blistering diseases that includes Pemphigus vulgaris (PV) and Pemphigus foliaceus (PF). Lesion morphology in PV and PF has been framed within the dictates of the desmoglein compensation hypothesis (DCH), an elegant hypothesis based on patient desmoglein-specific autoantibody profiles to predict lesion morphology. Central to this theory was the discovery of PV associated autoantibodies targeting the cadherin proteins Desmoglein-3 (Dsg3) and Desmoglein-1 (Dsg1), key components of desmosomal cell-cell attachments critical for epidermal integrity (1). According to the DCH, differences in lesion morphology in PV and PF are attributable to differential expression of the Dsg1 and Dsg3 proteins throughout the epidermis. Dsg1 is expressed at higher concentrations towards the superficial layers of the skin and mucosal epidermis, while Dsg3 is concentrated towards the basal layers. Non-mucosal skin notably expresses higher levels of Dsg1 throughout the epidermal layers, while Dsg3 is only present in the basal layers. Conversely, the mucosal membranes have higher concentrations of Dsg3 throughout the epidermis while Dsg1 is present in the superficial layers of the epidermis only.

The DCH posits that the differential expression of Dsg3 and Dsg1 proteins in mucosal vs. non-mucosal skin underlies the clinical presentation of 3 distinct classes of lesion morphology in Pemphigus: 1) PF is characterized by subcorneal skin dissociation, and is linked to the presence of only anti-Dsg1 autoantibodies. 2) Mucosal PV presents with suprabasilar blistering, hypothesized to be a result of only anti-Dsg3 autoantibodies. 3) Mucocutaneous PV is characterized by suprabasilar skin and mucosal lesions and is attributed to the presence of anti-Dsg1 and anti-Dsg3 autoantibodies (2).

While the DCH attempts to account for the pathology and clinical presentation in PF as well as mucosal and mucocutaneous PV, it cannot explain the cutaneous only manifestation of PV (cPV), where patients present with suprabasilar lesions on non-mucosal skin, including those that do not have *any history of* mucosal lesions. Yet, the cPV phenotype has been reported in several case studies (3–5). Currently, there are a limited number of reported cases of cPV and, upon review of the literature, most of these cases have been in Japanese patients. There have been only two reported Caucasian patients with cPV (6, 7). While no individual cases of cPV in the United States have been reported in literature, analysis of PV patient data collected in a registry initiated by the International Pemphigus and Pemphigoid Foundation revealed that 24.5% of patients reported only cutaneous lesions at the time of clinical screening (8).

A distinction that is often not made when assessing cPV is the patient’s lesion history, namely if they have had mucosal lesions in the past, or if they have only ever presented with cutaneous lesions. One example where this information is noted is Nagasaka et al., 2005, in which the patient’s lack of mucosal lesion history is explicitly stated (3). Thus, we note the distinction of two cPV subgroups: cPV with history of mucosal lesions (cPVwhm), and cPV without history of mucosal lesions (cPVwohm). Here, we identify 3 biopsy confirmed PV patients with cPVwohm. We further examine the HLA type and anti-Dsg3 and anti-Dsg1 titers of these patients.

## Materials and methods

Participants were recruited to the study from the Dermatology outpatient clinics at the University at Buffalo, Weill Cornell Medical College, and Michigan State University, as well as annual meetings hosted by the International Pemphigus and Pemphigoid Foundation (IPPF) for enrollment to our autoimmune blistering disorder biorepository. The study was approved by the institutional review boards of the respective academic institutions and followed all ethical guidelines, including written informed consent prior to study enrollment.

Patients provided information about their demographics, disease history, disease classification, lesion morphology, past medical history, and family history. Venous blood samples were collected from which serum was isolated and used immediately or stored at -80°C for future analysis. Patients with more than 1 visit provided venous blood and current clinical information at every visit when possible.

Of a total of 408 PV patients analyzed for this study, 34 patients reported a biopsy-confirmed case of PV and presented with active cutaneous lesions only (cPV) at time of enrollment. These patients, while not featured in this report, are presented in the context of a larger study presenting PV cases that violate the postulates of the DCH (Sielski et al.).

Of these patients, 7 reported no history of mucosal lesions. Three of those 7 patients were able to provide biopsies confirming their diagnosis of PV as well as confirm that they did not have a history of mucosal lesions. Patients were considered active if they met the criteria of 3 or more non-transient lesions and/or extension of current lesions in the past month (9). For the purposes of this study, the diagnosis of PV was determined by histopathological findings (suprabasilar acantholysis) and DIF (IgG and C3 deposition in intercellular epidermis).

High resolution HLA typing was performed *via* PCR amplification using sequence specific primers at the Histocompatibility and Immunogenetics Laboratory at Michigan State University employing commercial kits (One lambda, Thermo Fisher Scientific) (10). Patients were deemed “HLA-positive” if they possessed one or both of the PV-associated HLA alleles, DRB1*0402 and DQB1*0503. Patients that did not have either of these alleles were considered “HLA-negative”.

Anti-Dsg 3 and -1 levels were detected by ELISA (MBL Intl. (RG-M7593-D) as per manufacturer’s protocol with a 1:101 serum dilution. These kits detect immunoglobulin G (IgG) antibodies directed against Dsg1 and Dsg3, but do not distinguish between IgG subclasses. Antibody positivity was defined as ELISA levels of >20U/mL for both anti-Dsg1 and anti-Dsg3. The cutoff of 20U/mL was used as it was previously used by the manufacturer and has been used as a cutoff in other studies into PV (11).

## Results

Patient 1 is a 64-year-old male of South Asian descent. This patient initially presented with eroded lesions on the scalp. He was not receiving any immunotherapy at the time of presentation. His biopsy showed suprabasilar acantholysis, with DIF showing intraepidermal separation and weak IgG and C3 deposition in the intracellular epidermis consistent with a diagnosis of Pemphigus vulgaris. A blood draw two weeks after the initial biopsy revealed elevated anti-Dsg1 (61 U/ml), but not anti-Dsg3 levels. Additionally, the patient carries a known PV-associated HLA risk allele, DQB1*0503. Over the course of the past 6 years and as of writing of this report, the patient has never experienced mucosal lesions (**Table 1** **)**.

**Table 1:** Patient Demographics and Clinical Information.

	Patient 1	Patient 2	Patient 3
Patient ID	PV 386	PV 445	PV 463
Sex	Male	Male	Female
Ethnicity	South Asian	Caucasian	Caucasian
Age of onset	64	68	59
**HLA-Association**			
DRB1	13:01 and 14:04	16:01 and 16:02	03:01 and 16:01
DQB1	**0503** and 06:03	05:02 and 15:02	02:01 and 05:02
HLA status	“Positive”	“Negative”	“Negative”
**Biopsy Findings**			
Date of Biopsy	Oct. 1, 2014	Feb. 8, 2017	Dec. 27, 2016
Histology	**Suprabasilar** Acantholysis	Acantholysis in mid-upper epidermal layer.	**Suprabasilar acantholysis** and tombstoning
Direct Immuno-fluorescence (DIF)	DIF shows perilesional skin with intraepidermally separated epidermis. Weak IgG and C3 deposits in intercellular epidermis	DIF distributed along **lower 3/4 of epidermal thickness**. Antibodies to IgG and C3 show intercellular deposits	IgG and C3 intercellular distribution
Date of Repeat Biopsy		Nov. 19, 2019	
Histology		**Suprabasilar** and intergranular **acantholysis** with scale crust and parakeratosis consistent with pemphigus	
**Serum Antibodies and Clinical Information**			
Date of Blood Draw	Oct. 15, 2014	May 8, 2017	Oct. 13, 2018
Anti-DSG1	**61 U/ml**	3.7 U/ml	4.7 U/ml
Anti-DSG3	0.8 U/ml	1.5 U/ml	0 U/ml
Lesion Morphology at Time of Draw	Eroded lesions on scalp, one quadrant	2 blisters above waist, 1 on right tricep, 1 on leg, 1 on left shoulder	Multiple lesions on back, arms, and legs
Activity PDAI at Time of Draw	1	4	5
Medications at Time of Draw	No immunotherapy	Mycophenolate 1g BID, rituximab infusion 3 weeks ago, 40 mg prednisone/day, IVIG infusion 2 weeks ago	Mycophenolate 1g/day, rituximab infusion 2 months ago

Relevant data include Histological and DIF finding, serum autoantibodies and associated morphology at time of blood draw. “HLA Positive” describes a carrier of the PV -susceptibility alleles DRB1*0402 and/or DQB1*0503; “HLA Negative” describes a subject that does not carry either of the PV -susceptibility alleles DRB1*0402 and/or DQB1*0503.Certain values were bolded in Table 1 in order to draw the attention of the reader to some of the pertinent findings.

**Figure 1:**
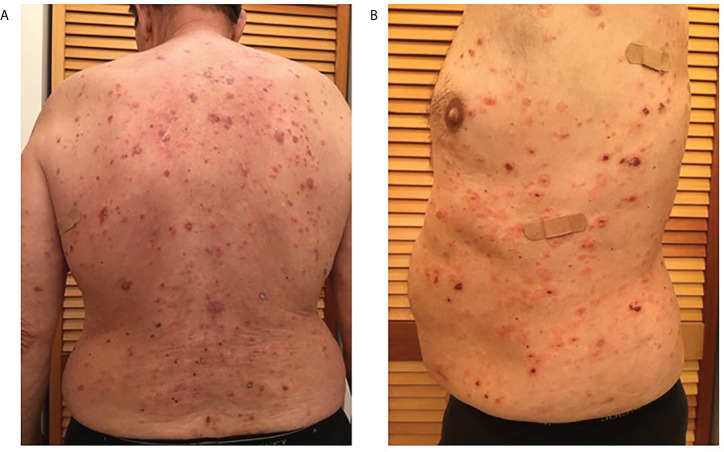
Multiple scattered blisters and erosions in various stages of healing observed over the entire torso **(A.** back, and **B.** left side) of patient 2 prior to original biopsy (February 2017).

Patient 2 is a 68-year-old male of Caucasian descent. He presented with blisters on the waist, legs, and both arms (**Figure 1**). His initial biopsy showed acantholysis in the mid-upper epidermal layer. DIF revealed IgG and C3 intercellular deposits along the lower aspects of the epidermal layer. A later biopsy showed suprabasilar and intragranular acantholysis. He did not carry either of the reported PV-associated HLA alleles DRB1*0402 or DQB1*0503. Upon presentation to our group 3 months after the initial biopsy, the patient had received rituximab (1g i.v. infusion 3 weeks and 5 weeks prior), and IVIg infusion (2g/kg over 3 days; 2 weeks prior) and was on mycophenolate mofetil 1g BID and, 40mg prednisone daily, but was still presenting with active disease (erosions on torso and extremities). Anti-Dsg1 antibodies recorded at low levels (3.7U/ml respectively) but well below the manufacturer-set cutoff for positivity in this patient presenting in active disease. There is no documented history of mucosal lesions (**Table 1** **)**.

Patient 3 is a 59-year-old female of Caucasian descent. Her diagnostic biopsy revealed suprabasilar acantholysis and tombstoning consistent with PV. Her DIF also showed intercellular deposition of IgG and C3. She did not type for either PV associated risk alleles, DRB1*0402 or DQB1*0503. Upon intake and blood draw, roughly 2 years after her initial diagnosis, she presented with multiple skin lesions on her back, arms, and legs, without history of mucosal disease. At the time of evaluation, she was receiving mycophenolate mofetil 1g/daily, and her last rituximab infusion (1g x 2, 2 weeks apart) was 2 months prior. Despite showing disease activity with a PDAI of 5, her anti-Dsg3 was undetectable at 0U/ml and anti-Dsg1 was recorded well below the cutoff for positivity at 4.7 U/ml (**Table 1** **)**.

## Discussion

Pemphigus encompasses a group of autoimmune blistering disorders in which the precise location of epidermal splitting is key to accurate diagnoses. The DCH categorizes patients into one of three groups based on the level of the intradermal split (suprabasilar acantholysis in the mucosa for mucosal PV, suprabasilar acantholysis in skin and mucosa for mucocutaneous PV, and subcorneal acantholysis in nonmucosal skin only for PF), but does not predict the existence of PV patients with cutaneous lesions in the absence of mucosal lesions (1, 4, 8, 12). A cutaneous only expression of PV (cPV) as reported in the literature, (**Table 2**), including those patients without any history of mucosal lesions (cPVwohm), cannot be accommodated within the current paradigms. Modified models of disease will be required to better account for the full spectrum of clinical presentation in pemphigus.

**Table 2:** Cases of cutaneous only PV reported in the literature.

Cases	Age (y)	Sex	Skin Lesion	Mucosal Lesion	Duration of cPV	Phenotype	Supra-basilar acantholysis	Superficial acantholysis	Skin DIF	Anti-Dsg1 (index values)	Anti-Dsg3 (index values)
Müller et al. (2002) (7)											
*#1*	51	M	Positive	−	ND	cPV	Positive	Positive	Positive	192.2	31.1
Yoshida et al. (2005) (4)											
*#2*	52	F	Positive	−	3 mo	cPV	Positive	−	Positive	2160	301.8
*#3*	52	M	Positive	−	10 y	cPV	Positive	Positive	Positive	459.6	82.5
*#4*	58	F	Positive	−	8 mo	mcPV-R-cPV	Positive	−	Positive	651.6	462.8
*#5*	57	F	Positive	−	7 mo	cPV	Positive	−	Positive	140.2	90.6
Nagasaka et al. (2005) (3)											
*#6*	45	F	Positive	−	ND	cPV	Positive	−	Positive	114	42
Shinkuma et al.(2008)(5)											
*#7*	50	M	Positive	−	2 weeks	cPV	Positive	Positive	Positive (skin and oral mucosa)	680	220
Bello et al. (2013) (12)											
*#8*	30	M	Positive	−	5 months	cPV	Positive	–	Positive	Not Assessed	Not Assessed
Carew et al. (2014) (6)											
*#9*	79	M	Positive	−	6 weeks	cPV	Positive	−	Positive	10	160

A total of nine cases of cPV have been reported to date between 2002-2014. With the exception of Nakasaka et al., no other study documents whether mucosal lesions had been present in the past. Studies have used varying criteria when reporting cPV, including one patient (Patient #3) who displayed both suprabasilar acantholysis (diagnostic of PV) and superficial acantholysis (diagnostic of PF) (4). ND, Not Determined; cPV, Cutaneous type Pemphigus Vulgaris; mcPV, mucocutaneous Pemphigus Vulgaris; R, Remission.

We report here 3 additional cases of cPV that are at odds with the postulates of the DCH which predicts suprabasal blistering in the skin only when accompanied by lesions in the oral mucosa. These are the first individually reported cases of cPV in North America, as well as examples of the subgroup cPVwohm, presenting with no history of mucosal lesions. One limitation faced in diagnosing this subgroup is the reliance on patient provided data. Many patients face delays in diagnosis making it potentially difficult for individuals to accurately recall isolated mucosal lesions, despite their strong assertions regarding the extent of their clinical lesions along with extensive interrogation of their clinical history for this study by trained clinical investigators (8). It should also be noted that while Patient 1 was classified as biopsy-proven pemphigus vulgaris with suprabasilar acantholysis, his lesions on presentation were generally superficial erosions and he had elevated anti-Dsg1 levels without anti-Dsg3 antibodies. As such, it cannot be ruled out that this case could represent atypical PF rather than atypical PV (in a patient of South Asian descent carrying the PV-associated HLA DQB1*0503 allele with a predominantly cutaneous profile as discussed below). In any case, this patient illuminates the complexities of assigning subtypes of pemphigus, and reinforces the point that our current diagnostic paradigms for PV and PF may be limited and oversimplified under the stricture of the DCH as currently formulated.

A strong association with the HLA-alleles DRB1*0402 and DQB1*0503 has been shown for PV, with the vast majority of Caucasian and Ashkenazi Jewish patients carrying one or both of these alleles (13–15). Only one of the three cPV patients reported in this study carried the known PV-associated HLA risk allele DQB1*0503 and presented with an elevated anti-Dsg1 antibody level. This patient’s presentation is in line with reports in the literature of PV patients of South Asian descent carrying DQB1*0503 as well as having higher levels of anti-Dsg1 and higher rates of cutaneous disease (16). Of note, two of the three patients presented here did *not* carry the primary PV-associated HLA alleles, and thus may be carriers of non-Dsg antibodies such as anti-desmocollin or others. These same two patients did not meet the threshold for positivity for the key PV associated autoantibodies, anti-Dsg3 and anti-Dsg1, despite being in the active phase of disease at the time of evaluation. While the two patients did have some level of detectable anti-Dsg1 (albeit well below the manufacturer-set level of detectability), this could reflect residual autoantibodies in a previously antibody positive patient, or represent highly reactive autoantibodies. We acknowledge that in these cases serum was sampled after treatment with the anti-CD20 antibody Rituximab (either 3 weeks or 2 months prior to blood draw) which has been shown to lead to a reduction in anti-desmoglein antibodies (17). Nonetheless, even without detectable anti-Dsg3 and anti-Dsg1 antibodies, both patients still presented with active lesions (PDAI score of 4 and 5, respectively) and showed IgG and C3 deposition by DIF, consistent with the diagnosis of PV, but also at odds with the DCH. While anti-Dsg3 and anti-Dsg1 autoantibodies are believed to be the primary mediators of lesional activity in PV, the literature does include studies reporting patients with biopsy proven PV in active disease with negative autoantibody levels (18). These findings lend support for the possibility that other factors, potentially including non-desmoglein autoantibodies, are relevant for blister formation in PV patients, perhaps particularly in cPV cases (19–21). Interestingly, for patient 2, the DIF finding of antibody distribution “along the lower ¾ of epidermal thickness” may indicate the presence of non-Dsg antibodies with a distribution in those areas such as antibodies against desmocollin 2 or -3 (22).

The HLA negative status of 2/3 cPV patients reported here along with the absence of anti-Dsg3 and anti-Dsg1 antibodies suggests that differences in HLA genetics may be linked to distinct autoantibody profiles, that are in turn linked to variance in clinical presentation (mucosal PV vs. mucocutaneous PV vs. cutaneous PV). Of note, the majority of cPV cases reported in the literature are from Japan, a population in which Pemphigus is not as tightly linked to the PV-associated HLA alleles DRB1*0402 and DQB1*0503 (23). The further exploration of the genetic and immune profiles of uncommon PV clinical subgroups such as cPV patients, including cPVwohm, can be expected to deepen our understanding of disease risk, disease mechanisms and disease presentation, and ultimately allow us to better predict, prognose and manage the full range of pemphigus cases.

## Data availability statement

The original contributions presented in the study are included in the article/supplementary material. Further inquiries can be directed to the corresponding author.

## Ethics statement

The studies involving human participants were reviewed and approved by Institutional Review Board, Jacobs School of Medicine and Biomedical Sciences; Institutional Review Board, Michigan State University; Institutional Review Board, Weill Medical College of Cornell University. The patients/participants provided their written informed consent to participate in this study. Written informed consent was obtained from the individual(s) for the publication of any potentially identifiable images or data included in this article.

## Author contributions

JB, KS-S, and AS devised the project. KS-S and AS collected patient samples and associated clinical data. JB performed the analysis, and drafted the first version of the article. KS-S and AS performed critical revision of the manuscript.

## Funding

This study was supported by internal departmental funding.

## Conflict of interest

The authors declare that the research was conducted in the absence of any commercial or financial relationships that could be construed as a potential conflict of interest.

## Publisher’s note

All claims expressed in this article are solely those of the authors and do not necessarily represent those of their affiliated organizations, or those of the publisher, the editors and the reviewers. Any product that may be evaluated in this article, or claim that may be made by its manufacturer, is not guaranteed or endorsed by the publisher.
